# Genes as Cues of Relatedness and Social Evolution in Heterogeneous Environments

**DOI:** 10.1371/journal.pcbi.1005006

**Published:** 2016-06-24

**Authors:** Olof Leimar, Sasha R. X. Dall, Peter Hammerstein, John M. McNamara

**Affiliations:** 1 Department of Zoology, Stockholm University, Stockholm, Sweden; 2 Centre for Ecology and Conservation, University of Exeter, Penryn, United Kingdom; 3 Institute for Theoretical Biology, Humboldt University Berlin, Berlin, Germany; 4 School of Mathematics, University of Bristol, Bristol, United Kingdom; CNRS, FRANCE

## Abstract

There are many situations where relatives interact while at the same time there is genetic polymorphism in traits influencing survival and reproduction. Examples include cheater-cooperator polymorphism and polymorphic microbial pathogens. Environmental heterogeneity, favoring different traits in nearby habitats, with dispersal between them, is one general reason to expect polymorphism. Currently, there is no formal framework of social evolution that encompasses genetic polymorphism. We develop such a framework, thus integrating theories of social evolution into the evolutionary ecology of heterogeneous environments. We allow for adaptively maintained genetic polymorphism by applying the concept of genetic cues. We analyze a model of social evolution in a two-habitat situation with limited dispersal between habitats, in which the average relatedness at the time of helping and other benefits of helping can differ between habitats. An important result from the analysis is that alleles at a polymorphic locus play the role of genetic cues, in the sense that the presence of a cue allele contains statistical information for an organism about its current environment, including information about relatedness. We show that epistatic modifiers of the cue polymorphism can evolve to make optimal use of the information in the genetic cue, in analogy with a Bayesian decision maker. Another important result is that the genetic linkage between a cue locus and modifier loci influences the evolutionary interest of modifiers, with tighter linkage leading to greater divergence between social traits induced by different cue alleles, and this can be understood in terms of genetic conflict.

## Author Summary

The theory of kin selection explains the evolution of helping when relatives interact. It can be used when individuals in a social group have different sexes, ages or phenotypic qualities, but the theory has not been worked out for situations where there is genetic polymorphism in helping. That kind of polymorphism, for instance cheater-cooperator polymorphism in microbes, has attracted much interest. We include these phenomena into a general framework of social evolution. Our framework is built on the idea of genetic cues, which means that an individual uses its genotype at a polymorphic locus as a statistical predictor of the current social conditions, including the expected relatedness in a social group. We allow for multilocus determination of the phenotype, in the form of modifiers of the effects of the alleles at a cue locus, and we find that there can be genetic conflicts between modifier loci that are tightly linked versus unlinked to the cue locus.

## Introduction

Traditional theories of social evolution in structured populations use reproductive value to describe the fitness effects of variation in helping and harming traits [1–4]. They are applied to population structures such as the two sexes [1], juveniles and adults [3], dispersers and non-dispersers [5], and high- and low-quality individuals [4]. Individuals can, depending on their state, vary in their phenotype, which corresponds to a reaction norm [4], but genetic polymorphism in social traits is not explicitly included in the theory. Although it is recognized that frequency dependence is compatible with social evolution theory [6], questions of the emergence and maintenance of genetic polymorphism in social traits have not been given full attention. This absence is striking, as the possibility of such genetic polymorphism has attracted much interest. Examples of studies in the laboratory and the field span from work on cheater-cooperator polymorphisms [7–15] to investigations of genetic variation in microbial pathogens [16, 17]. The possibility that population structure contributes to polymorphism also has support [18–22].

It is already well understood that a social trait, such as an individual’s investment in helping, can evolve to different equilibria depending on the relatedness in social groups in different habitats, with more helping in habitats where there is higher relatedness. We use the concept of genetic cues to extend this insight to situations where there is dispersal between habitats and where the social trait is influenced by several, linked or unlinked, genetic loci. The basic idea of genetic cues [23–26] is that alleles can function as statistical predictors of coming selective conditions for an individual. As a consequence of selection, allele frequencies can differ between local environments, such that possessing particular alleles correlates with local conditions in a manner analogous to environmental cues. Using this insight one can integrate genetic polymorphism into theories of conditional phenotype determination.

If the environmental heterogeneity includes characteristics that are important for social evolution, like the size or composition of social groups, the heterogeneity could favor genetic polymorphism in social traits. If so, there will be a correlation between gene frequencies and social characteristics, and genes can act as cues of relatedness. To illustrate this general idea we develop a specific model with two habitats. We show that alleles at a cue locus can provide information about social circumstances, such as within-group relatedness and opportunities for cooperation, and that epistatic modifiers of the phenotypic effects of a genetic polymorphism can evolve to make use of this information. We also show that the evolutionary interests of epistatic modifiers can differ depending on their degree of linkage to a polymorphic locus, and we interpret this phenomenon in terms of genetic conflict.

## Model

There are two habitats, each containing a large number of groups. They are formed and dissolved by colonization followed by social interaction and the production of offspring that disperse, and again colonization. A group in habitat *i*, where *i* = 1, 2, is founded by *N*_*i*_ haploid individuals, randomly derived from a pool of dispersers in that habitat. To implement variation between habitats in average within-group relatedness, group members reproduce asexually following founding, forming *N*_*i*_ haploid offspring group members, such that each founding group member has an equal and independent chance of producing each of the *N*_*i*_ offspring (model details are given in S1 Text). A smaller *N*_*i*_ thus corresponds to higher relatedness. For a pair of group members, the probability of being identical by descent since founding is

$$r_{i}=\frac{1}{N_{i}}\text{,}$$ (1)

which follows [27] and [28]. The offspring group members engage in a social interaction, for instance a public goods game [29], and produce dispersing offspring in proportion to the payoff in the game. An individual’s phenotype *z* represents an investment (strategy) in the game, and we assume 0 ≤ *z* ≤ 1. The payoff to an individual with phenotype *z* in habitat *i* is a function $w_{i}\left(z,\bar{z}\right)$ of *z* and the average investment $\bar{z}$ of the individual’s group. As a convenient example we will use $w_{i}\left(z,\bar{z}\right)=W_{i}+b_{i}\bar{z}-c_{i}z^{2}$, where the benefit $b_{i}\bar{z}$ is proportional to the average investment and the cost *c*_*i*_ *z*^2^ is assumed to increase quadratically with the individual’s investment. For polymorphic populations the group compositions will vary, and we are particularly interested in the expected payoff in habitat *i* to a randomly chosen rare mutant player of the game with phenotype *z*′, in a population where the resident phenotypes *z*_1_ and *z*_2_ occur with frequencies *p*_*i*1_ and *p*_*i*2_ (where *p*_*i*1_ + *p*_*i*2_ = 1). We write this as

$${\bar{w}}_{i}^{\prime }=E\left[w_{i}\left(z^{\prime },\bar{z}\right)|z_{1},z_{2},p_{i1},p_{i2}\right]\text{.}$$ (2)

**Fig 1:**
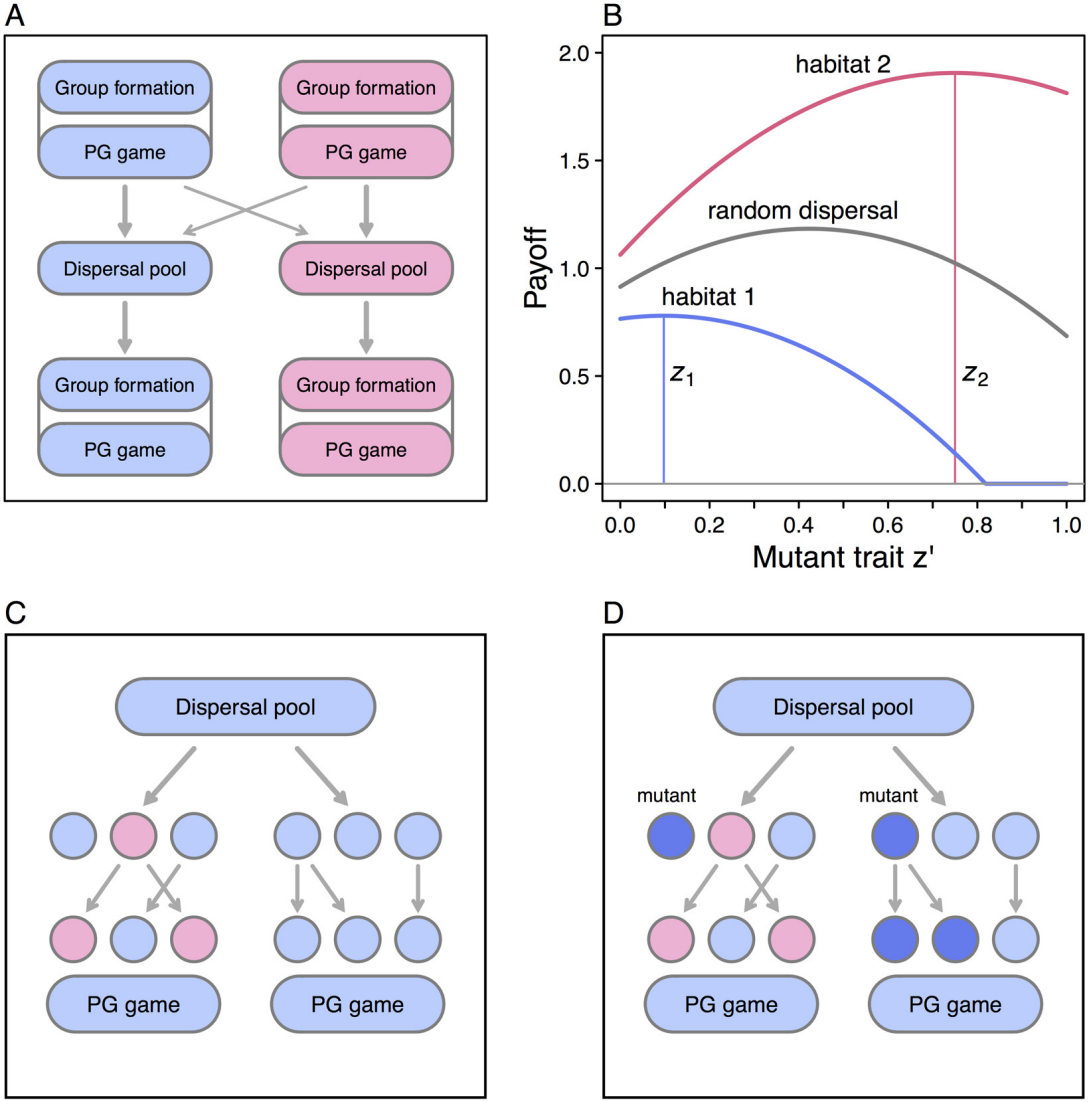
Elements of the model. Panel (A) shows the population cycles in habitat 1 (color coded blue) and habitat 2 (red), including formation of social groups and playing the public goods game, resulting in the production of dispersing offspring, some of which go to the dispersal pool in their birth habitat and some go the pool in the other habitat. New social groups are then formed from the pool in each habitat. (B) The expected payoff Eq (2) for mutant trait *z*′ in habitat 1 (blue) and habitat 2 (red) in the limit of no between-habitat dispersal. The resident traits in habitats 1 and 2 are *z*_1_ and *z*_2_ (blue and red vertical lines). The gray curve shows mutant payoff when there is random dispersal, with the same two resident traits. (C) Illustration of group formation for two groups in habitat 1 with *N*_1_ = 3. First founding group members are randomly drawn from the dispersal pool, followed by asexual reproduction forming *N*_1_ offspring, each of which is a copy of a randomly selected parent in the founding group. (D) For a rare mutant (darker blue), founding groups with mutants will predominantly contain a single mutant. The offspring groups can contain from 0 to *N*_1_ mutants, and in expectation contain one mutant.

Because a new group is founded by random dispersers, those groups containing mutant strategies will predominantly be founded by one mutant and *N*_*i*_ − 1 resident types. Some basic aspects of the model are illustrated in Fig 1.

To study the invasion of mutant traits, we need the derivative of the expected mutant payoff, which we write as

$$d_{ik}={\left.\frac{\partial {\bar{w}}_{i}^{\prime }}{\partial z^{\prime }}\right|}_{z^{\prime }=z_{k}}=\frac{b_{i}}{N_{i}}\left(1+\left(N_{i}-1\right)r_{i}\right)-2c_{i}z_{k}\text{,}$$ (3)

for habitat *i* and phenotype *z*_*k*_, *i* = 1, 2, *k* = 1, 2. We study evolutionary change of a dimorphism *z*_1_, *z*_2_ by examining the invasion of mutant modifiers. Let *x*_1_ and *x*_2_ denote two alleles at the cue locus. In the resident population, the genetic cue *x*_*k*_ induces the phenotype *z*_*k*_, and *n*_*ik*_ is the number of individuals in habitat *i* with phenotype *z*_*k*_ at a population dynamical equilibrium. The epistatic effect of a mutant modifier is that *x*_*k*_ instead induces the phenotype $z_{k}^{\prime }$. Letting $n_{ik}^{\prime }$ denote the (small) number of mutant modifiers in habitat *i* with phenotype $z_{k}^{\prime }$ (i.e., linked to cue allele *x*_*k*_), we can write down a population projection matrix for the mutant invasion. The invasion fitness of the mutant modifier is

$$F(z_{1}^{\prime },z_{2}^{\prime };z_{1},z_{2})=\log\lambda \text{,}$$ (4)

where *λ* is the leading eigenvalue of the population projection matrix. Here we give an overview of the derivation of this matrix (details are given in S1 Text).

For simplicity, we assume that individuals are haploid over most of the life cycle. However, to explore the consequences of recombination between cue and modifier loci, we introduce sexual reproduction by assuming there is a brief sexual phase in the dispersal pool in a habitat. This involves diploid individuals and crossing over, with a recombination rate *ρ* between cue and modifier loci, to produce the haploid individuals that found the groups as described above. Mating is random with respect to the dispersal pool and occurs before the forming of groups in the habitat. As a census point, we specify the population composition at a time after the sexual phase, when groups have formed and the public goods game is about to start. The sequence of events in the life cycle, starting right after the census point, is as follows: (*i*) public goods game with offspring production in proportion to payoff, (*ii*) within- and between-habitat migration of these offspring, forming a dispersal pool in each habitat, (*iii*) mating and recombination, and (*iv*) the next episode of group formation, including one asexual generation. By putting these events together, one can write down the matrix (see S1 Text). Using the population dynamics we can also determine the region of coexistence of two phenotypes *z*_1_ and *z*_2_ for different sets of parameters, by determining when each phenotype can invade a monomorphism of the other (the condition is given in equation (S16) in S1 Text).

We compute a selection gradient from the invasion fitness Eq (4) using standard methodology of matrix population models [30]. Because we average over the group compositions Eq (2), our analysis is consistent with the structured population approach to adaptive dynamics [31], and it can also be seen as a direct fitness methodology for social evolution theory [1, 3], also referred to as a personal fitness methodology [6].

In order to check our analytical results, and to illustrate the effects of genetic conflict between cue and modifier loci, we have run individual-based evolutionary simulations corresponding to our model assumptions. As a genotype-phenotype mapping in these simulations, we used a sigmoid function

$$z=\frac{1}{1+\exp\left(-a_{0}-a_{g}x\right)}$$ (5)

of a ‘liability’ *a*_0_ + *a*_*g*_*x*, where *x* is the effect of an allele at the genetic cue locus, and *a*_0_ and *a*_*g*_ are parameters that are genetically determined by modifier loci (details are given in S1 Text).

To compute evolutionary equilibria numerically, we developed a C++ program that follows a path of small steps through *z*_1_ *z*_2_–space, each of which increase the invasion fitness Eq (4), until reaching an equilibrium. We used the Eigen C++ library [32] to compute eigenvalues. For the individual-based evolutionary simulations, we developed C++ programs that directly implemented the sequence of events in the life cycle, using pseudo-random numbers to handle stochastic events, such as recombination and mutation. In the simulations, we used a total populations size of 40 000 and time periods of 40 000 full life cycles or more.

## Results

### Selection gradient

We use the methodology of adaptive dynamics and matrix population modeling [30, 33] to compute the derivative of invasion fitness for a mutant modifier. The details of the derivation are given on pp. 8–10 of S1 Text, and here we focus on the interpretations in terms of information in a cue. The genetic cue provides information to an individual about its current habitat. The prior probability of being in habitat *i* is *q*_*i*_ = *n*_*i*_/(*n*_1_ + *n*_2_), where *n*_*i*_ = *n*_*i*1_ + *n*_*i*2_ is the number of individuals in habitat *i* at a population dynamical equilibrium and *n*_*ik*_ is the number of individuals in habitat *i* with phenotype *z*_*k*_. For an allele at a modifier locus, the probability of being in habitat *i*, conditional on being linked to allele *x*_*k*_ at the cue locus is

$$q_{ik}=\frac{p_{ik}q_{i}}{p_{1k}q_{1}+p_{2k}q_{2}}=\frac{n_{ik}}{n_{1k}+n_{2k}}\text{,}$$ (6)

where *p*_*ik*_ = *n*_*ik*_/*n*_*i*_. The selection gradient is the derivative of invasion fitness Eq (4) with respect to mutant traits, and can be written

$${\left.\frac{\partial F}{\partial z_{k}^{\prime }}\right|}_{z_{k}^{\prime }=z_{k}}=V_{1k}d_{1k}p_{k}q_{1k}+V_{2k}d_{2k}p_{k}q_{2k}\text{.}$$ (7)

To interpret this expression, note that *q*_1*k*_ and *q*_2*k*_ are the respective probabilities of being in habitat 1 or 2, conditional on being linked to cue allele *x*_*k*_. The factor *p*_*k*_ = (*n*_1*k*_ + *n*_2*k*_)/(*n*_1_ + *n*_2_) is a ‘dilution factor’ that appears because the mutant $z_{k}^{\prime }$ is only expressed in individuals with cue allele *x*_*k*_. The *d*_1*k*_ and *d*_2*k*_ are the derivatives of the expected payoff Eq (2) in habitats 1 and 2 with respect to the mutant trait, and are given in Eq (3). Finally *V*_*ik*_ is the reproductive value of an offspring of a player in habitat *i* with cue allele *x*_*k*_. From the manner in which the conditional probability *q*_*ik*_ appears in the expression, we can conclude that the selection gradient describes changes in payoff to a ‘Bayesian decision maker at the modifier locus’. Eq (7) is an extension of the direct fitness approach of social evolution theory to situations with genetic polymorphism at the cue locus. Note that this selection gradient refers to the invasion of mutant modifiers, and not to the invasion of alleles at the cue locus, except for the special case of full linkage (*ρ* = 0), for which cue and modifier form a unit.

Completing the life cycle, through migration, mating and recombination, and group formation, we can express *V*_*ik*_ in terms of reproductive values *v*_*jl*_ at our census point:

$$V_{ik}=\;v_{11}\phi_{1}h_{11k}m_{1i}+v_{21}\phi_{2}h_{21k}m_{2i}+v_{12}\phi_{1}h_{12k}m_{1i}+v_{22}\phi_{2}h_{22k}m_{2i}\text{.}$$ (8)

Here, *m*_*ji*_ is the rate of migration from habitat *i* to *j*. The ‘cue inheritance’ is described by

$$h_{jlk}=\left(1-\rho \right)\delta_{lk}+\rho p_{jl}\text{,}$$ (9)

so that with probability 1 − *ρ* the cue allele is passed to offspring and with probability *ρ* the offspring receives its cue allele through recombination with a random individual in the dispersal pool. Finally, *ϕ*_*j*_ is the probability for an individual in the dispersal pool in habitat *j* to become a founding group member.

We must also examine whether or not polymorphism can be maintained at the cue locus. This needs to be investigated as a separate question, by determining when each of the phenotypes *z*_1_ and *z*_2_ can invade a monomorphism of the other. The condition for this is given in equation (S16) in S1 Text.

### Illustration

**Fig 2:**
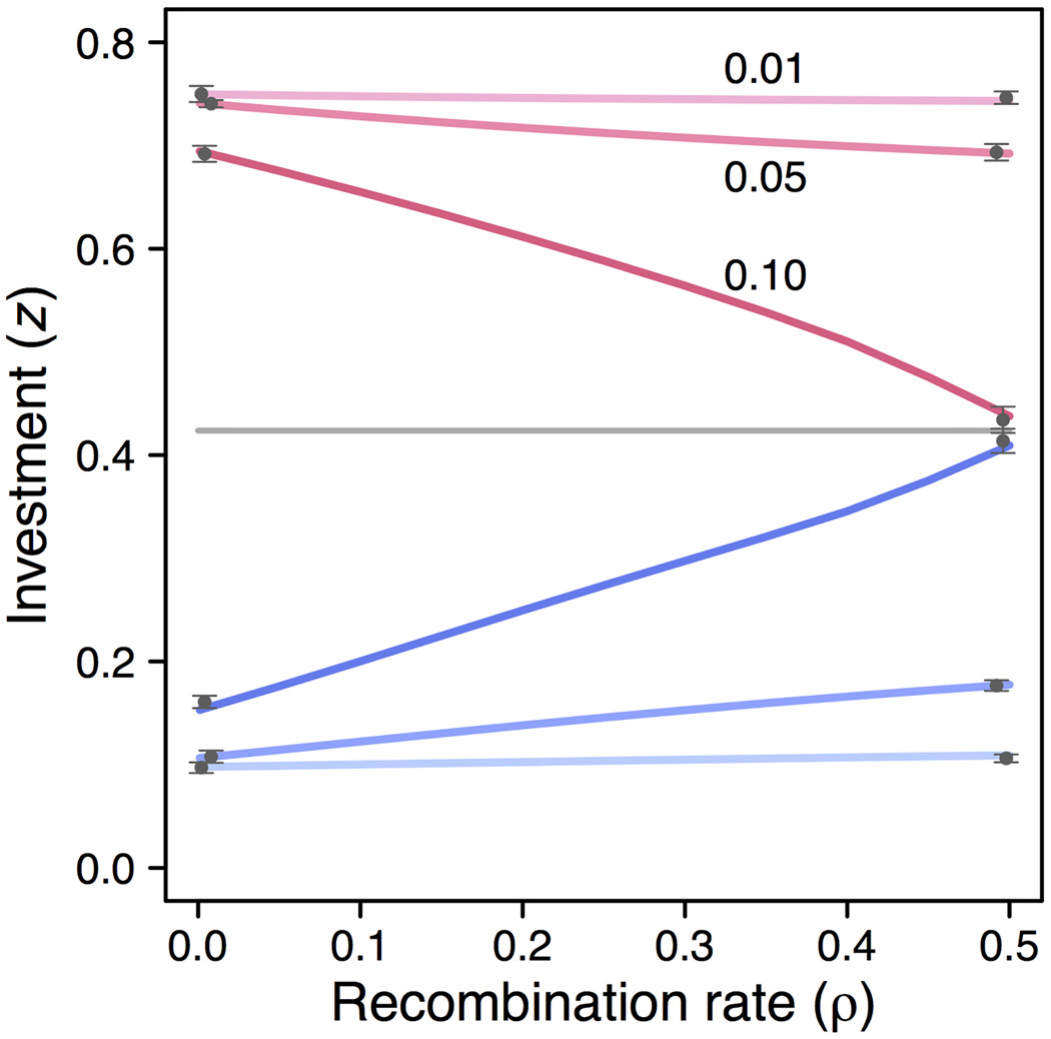
Evolutionary equilibrium dimorphisms. The equilibrium dimorphisms *z*_1_ and *z*_2_, color coded blue and red, are plotted as functions of the rate of recombination *ρ* between cue and modifier loci. The two habitats differ in the size of social groups, with *N*_1_ = 20 and *N*_2_ = 2, resulting in lower relatedness in habitat 1 (*r*_1_ = 0.05) than in habitat 2 (*r*_2_ = 0.5). Three examples are shown, labeled with the rate of migration between habitats: *m*_12_ = *m*_21_ = *m* = 0.01, 0.05, 0.10. The total population size is the same in both habitats, and the parameters for the public goods game are also the same: *W*_1_ = *W*_2_ = 0.5, *b*_1_ = *b*_2_ = 3.0, *c*_1_ = *c*_2_ = 1.5. The gray horizontal line shows the equilibrium of gradual evolution in a monomorphic population, which does not depend on *m* or *ρ*. The dark gray points (with error bars) at *ρ* = 0.0 and *ρ* = 0.5, shifted slightly left and right for visibility, show mean and standard deviation of the average phenotype over 10 replicate individual-based evolutionary simulations. In these simulations, *a*_*g*_ in Eq (5) was encoded by a single locus whereas *a*_0_ was kept at a fixed value (see S1 Text for further explanation).

**Fig 3:**
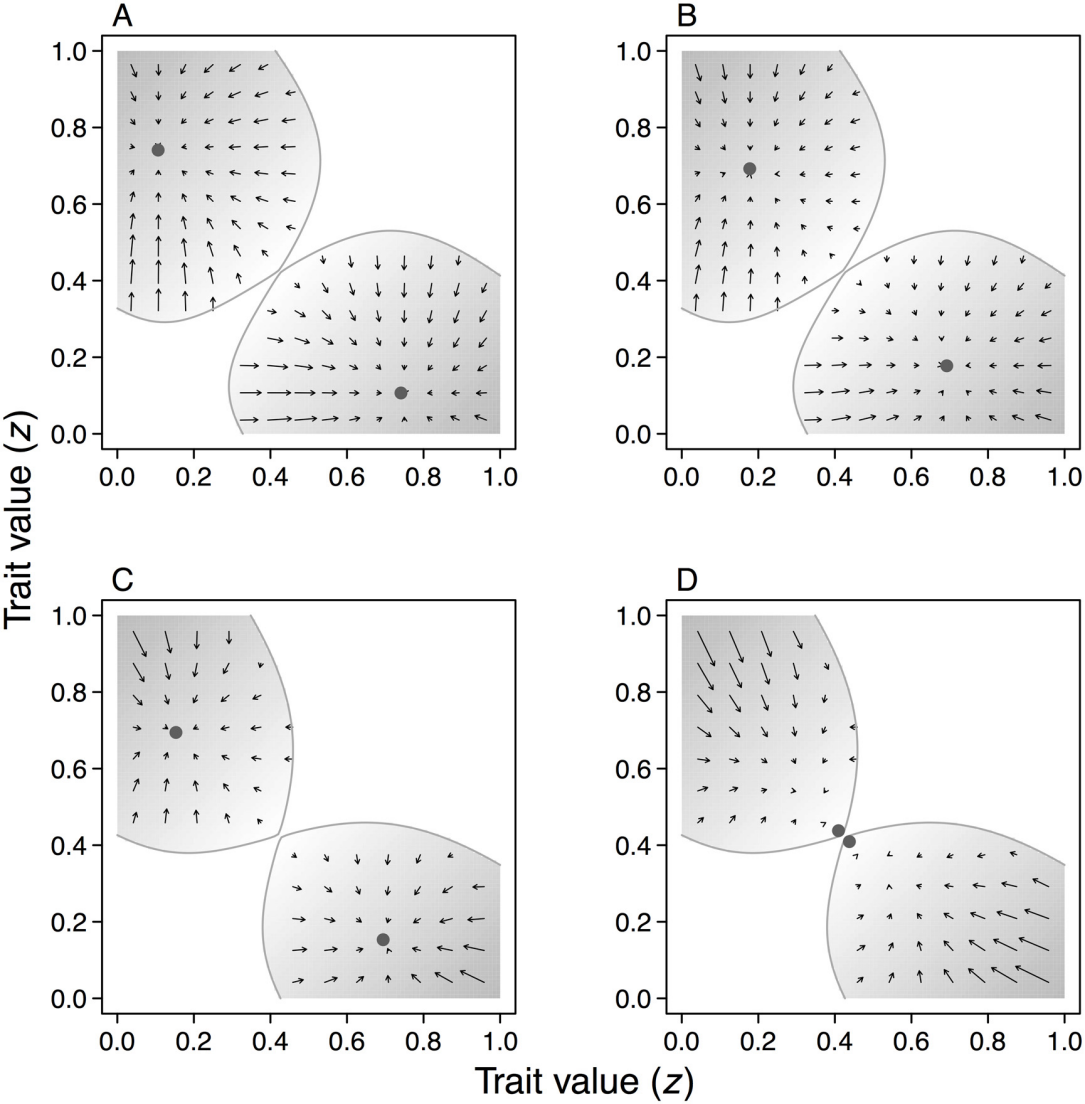
Trait evolution plots for dimorphisms. In each example, the shaded region shows where a dimorphism *z*_1_, *z*_2_ can be maintained, the arrows indicate the direction and magnitude of the selection gradient Eq (7), and the dots show evolutionarily equilibrium dimorphisms. The examples differ in between-habitat migration rate *m*_12_ = *m*_21_ = *m* and rate of recombination *ρ*. (A) *m* = 0.05, *ρ* = 0.001; (B) *m* = 0.05, *ρ* = 0.5; (C) *m* = 0.10, *ρ* = 0.001; (D) *m* = 0.10, *ρ* = 0.5. Other parameters: *N*_1_ = 20 and *N*_2_ = 2, *W*_1_ = *W*_2_ = 0.5, *b*_1_ = *b*_2_ = 3.0, *c*_1_ = *c*_2_ = 1.5.

Fig 2 shows how the migration rate *m* between habitats and the recombination rate *ρ* between cue and modifier loci influence dimorphic evolutionary equilibria, i.e. phenotypes where the selection gradient Eq (7) vanishes. The blue and red curves indicate phenotypes *z*_1_ and *z*_2_ suited to habitats with low and high relatedness. The selection gradient is illustrated in Fig 3 for a few values of *m* and *ρ*, and the shaded regions in this figure show where a polymorphism at the cue locus is maintained. In this example, the only difference between habitats is the number of founders of a social group, with *N*_1_ = 20 in habitat 1 and *N*_2_ = 2 in habitat 2, so it is appropriate to interpret the genetic cue as a cue of relatedness.

As seen in Fig 2, there is an interaction between the migration rate and the recombination rate, such that for very low migration rate (*m* = 0.01) the recombination rate has little influence on the equilibrium dimorphism, whereas for a higher migration rate (*m* = 0.10) the difference between *z*_1_ and *z*_2_ varies considerably from tight linkage to free recombination. For even higher rates of between-habitat migration, genetic polymorphism is not maintained at the cue locus, regardless of the cue-modifier recombination rate *ρ*, and the outcome is instead a monomorphism. For the parameter values in Fig 2, this happens for *m* = 0.15 or higher.

### Genetic conflicts

The divergence between *z*_1_ and *z*_2_ depends on *ρ*, as in Figs 2 and 3, because modifier alleles with different linkage to cue alleles have different demographic futures, and thus different evolutionary interests. A fully linked mutant modifier will remain more concentrated in one of the habitats, which tends to favor specialization to that habitat, whereas an unlinked one will fairly quickly become evenly distributed over cue alleles and habitats, which tends to favor less specialized phenotypes. This difference in evolutionary interest between modifiers follows the logic of genetic conflicts [34], in the sense that the invasion of a loosely linked modifier, reducing the divergence between phenotypes, creates the context for the invasion of a more tightly linked modifier that reverses this effect. The outcome of genetic conflicts can depend on such things as the of the availability of mutations, the genetic architecture of a trait, and the strength of selection.

For modifiers of polymorphic effects, genetic conflicts can have the further consequence of changing selection acting on the additive effects of alleles at a locus from stabilizing to disruptive, potentially giving rise to selectively maintained polymorphism at that locus. For instance, for the case of *m* = 0.10 in Fig 2, unlinked modifiers favor a very small divergence between *z*_1_ and *z*_2_, but once this outcome has been achieved, the selection on alleles at other loci with additive effects on *z* becomes disruptive (just as originally for the cue locus itself). Genetic polymorphism might then be transferred from an original cue locus to a new locus.

**Fig 4:**
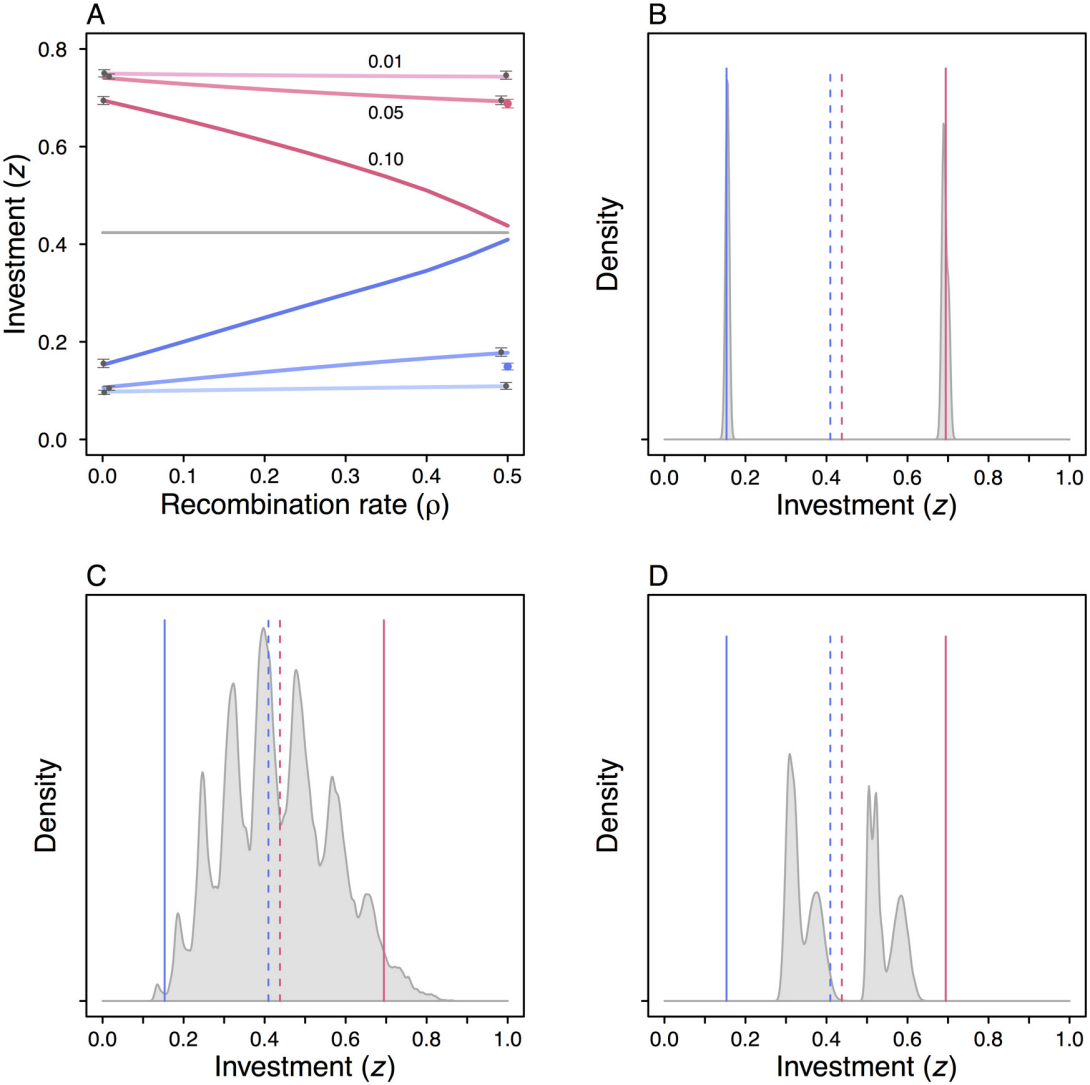
Results from individual-based simulations, illustrating consequences of genetic conflict. (A) Same as the simulations in Fig 2 except that *a*_0_, in addition to *a*_*g*_, in Eq (5) is determined by a single locus. The blue and red points (with error bars) show the deviating outcome for *m* = 0.10, *ρ* = 0.5, which is a consequence of genetic conflict between the cue locus and the locus encoding *a*_*g*_: *a*_*g*_ became close to zero, but *a*_0_ became polymorphic, and the polymorphism at the original cue locus collapsed. The outcome is further illustrated in (B), showing a kernel-smoothed distribution of phenotypes in a typical simulation. The blue and red vertical lines show the prediction from Fig 3C, where *ρ* = 0, and the blue and red dashed lines the prediction from Fig 3D, where *ρ* = 0.5. The outcome where an unlinked modifier (*a*_0_) takes over the polymorphism depends on the genetic architecture, as illustrated in (B), (C) and (D). In (C) the modifiers *a*_0_ and *a*_*g*_ in Eq (5) are each determined by several loci with small additive effects, and the loci contributing to *a*_0_ all became polymorphic. In (D) there is a more complex architecture for *a*_0_, with additive effects that in turn can be modified with an adjustable threshold limiting the amount of gene expression, and this threshold became polymorphic (see text and S1 Text for further explanation).

How this can happen is illustrated by the individual-based simulations in Fig 4. The genotype-phenotype mapping Eq (5) from the genetic cue *x* to the trait *z* has been changed from that in Fig 2, where the parameter *a*_0_ was fixed, to one where both parameters *a*_0_ and *a*_*g*_ are genetically determined and can evolve. In Fig 4A, *a*_0_ and *a*_*g*_ are each determined by a single locus, either fully linked or unlinked to each other and to the cue locus. When *m* is small or when *ρ* = 0, the outcome of the individual-based simulations remains in agreement with the predictions from the selection gradient Eq (7), but for *m* = 0.10 and *ρ* = 0.5, the outcome is instead the same as that for *m* = 0.10 and *ρ* = 0 (Fig 4B). The reason is that, starting with polymorphism at the cue locus, *a*_*g*_ evolved to become small, reducing the divergence between the phenotypes from Eq (5), which in turn gave rise to disruptive selection on *a*_0_, causing polymorphism to evolve at that locus, while the polymorphism at the original cue locus collapsed. The end result was that the locus coding for *a*_0_ became a polymorphic cue locus, with phenotypes *z*_1_, *z*_2_ in accordance with the evolutionary interests of fully linked modifiers of this new polymorphism (Fig 4B). Other conceivable evolutionary outcomes of disruptive selection on *a*_0_ are shown in Fig 4C and 4D.

**Fig 5:**
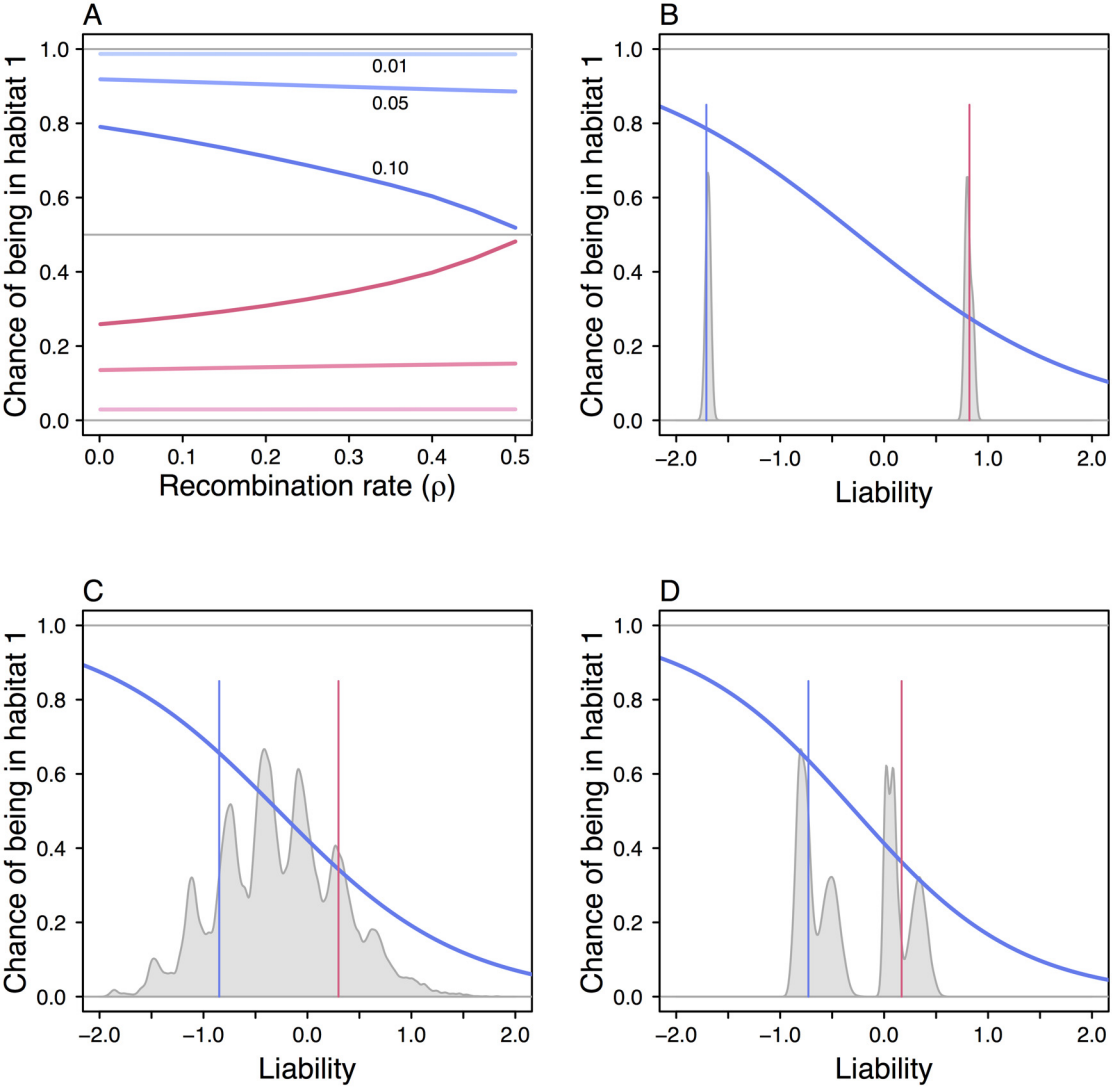
Illustration of the information contained in genetic cues. Panel (A) shows the conditional probability of being in habitat 1 (with *r*_1_ = 0.05) for an individual possessing cue allele *x*_1_ (blue curves, *q*_11_) versus cue allele *x*_2_ (red curves, *q*_12_). The probability is given as a function of the recombination rate *ρ* between cue and modifier loci. The three cases are from Fig 2, with different rates of between-habitat migration *m*_12_ = *m*_21_ = *m* = 0.01, 0.05, 0.10. The blue lines in panels (B) to (D) show logistic regressions of habitat 1 on the liability *a*_0_+*a*_*g*_ *x* in eq (5), for the individual-based simulations in Fig 4B to 4D (with *m* = 0.10 and *ρ* = 0.5). The distributions of this liability are shown in gray, and the vertical blue and red lines indicate ‘typical’ low and high values (mean ± sd for (C) and (D)). See S1 Text for further explanation.

In Fig 4C, 5 unlinked loci have small positive effects on *a*_0_ and 5 have small negative effects, and each of these loci became polymorphic in the simulation, while at the same time the original genetic cue locus remained polymorphic. The overall effect was a fairly broad distribution of values for the investment *z*. In Fig 4D, the maximum expression at the loci with positive and negative effects was controlled by two separate unlinked loci, and one of these became polymorphic, giving rise to a bimodal distribution of values of *z*. In these examples, a notable amount of genetic variation in *z* evolved, but the width and shape of the distribution of *z* depended on the details of the genetic architecture of the trait. In all cases, an individual gains information about its current habitat from its genotype, and one can show that the clearcut polymorphism in Fig 4B is the most informative, with progressively less information on average in Fig 4C and 4D, as illustrated in Fig 5. The latter cases are intermediate between the evolutionary interests of fully linked and unlinked modifiers.

## Discussion

We have shown how adaptively maintained genetic polymorphism can be integrated into social evolution theory by making use of the concept of genetic cues. The selection gradient we derived (in eq (7)) parallels the direct, or personal, fitness approach to social evolution in class-structured populations [3, 6], with the distinction that the presence of a cue allele in an individual’s genotype, rather than a phenotypic state, defines the class structure. In our model, individuals use strategies that are conditional on a genetic cue, but our general approach can incorporate a combination of genetic, environmental and transgenerational cues [26, 35].

Just as is the case in standard social-evolution theory, relatedness enters into our model as a description of the genetic structure of social groups. The structure refers to genetic variation at epistatic modifier loci, rather than at genetic cue loci, so the relatedness parameter *r*_*i*_ in the pay-off derivative Eq (3) refers to rare mutants at a modifier locus. This is in accordance with the general idea of treating genetic variation at a cue locus as input to a developmental or ‘decision-making’ system, and then to examine long-term evolution of the developmental system [24, 26]. The value of this perspective is that it guides the analysis and interpretation by forming a link to the study of conditional strategies, such as the study of phenotypic plasticity.

The different ways in which individuals gain information about themselves and their social partners has figured importantly in the study of social evolution [2]. For instance, migrant individuals arriving in a local population have different expectations of relatedness to their neighbors than non-dispersers [5]. The possibility that individuals can recognize kin through similarity in genetically polymorphic traits has been carefully investigated, with the conclusion that kin recognition can evolve in spatially structured populations [36–38]. Yet another possibility is that individuals estimate their degree of inbreeding, and thus how likely they are to be related to their neighbors, using their relative homozygosity as an indicator of local relatedness [39]. Our analysis of genetic cues of relatedness contributes to this general emphasis on the role of information, but differs from the other examples through an affinity to the study of local adaptation in the face of gene flow, which has been given much attention in evolutionary ecology but rather little in theories of social evolution.

We found that the genetic linkage between cue and modifier loci can influence the evolutionary outcome (Figs 2 and 3), and this gives rise to genetic conflicts. Genes unlinked to a genetic cue locus tend to favor phenotypes that are less specialized to particular habitats compared to tightly linked genes, because unlinked genes become adapted to exist in all habitats, be transferred between them, and to use the information in a genetic cue to adjust the phenotype in an optimal way for this situation. Tightly linked genes, on the other hand, might be selected to perform well in only one of the habitats, even at the expense of performance in another habitat. The reason is that a modifier allele tightly linked to a cue locus allele can become concentrated to one of the habitats, with the other habitat acting as a sink, to which little adaptation takes place [40, 41].

Our results on the role of genetic conflicts in giving rise to disruptive selection at modifier loci (Fig 4) extends the previous understanding of genetic conflicts when there is adaptively maintained genetic polymorphism [24]. Disruptive selection in heterogeneous environments can maintain genetic polymorphism [42], and genetic cue polymorphism is an example of this general phenomenon. So, if unlinked or loosely linked modifiers of a genetically polymorphic locus evolve to reduce or eliminate the divergence between phenotypes, there will be disruptive selection at loci with additive effects on the phenotype in question. Theoretical modeling has found that disruptive selection tends to favor genetic architectures where polymorphism is concentrated to a single locus [23, 43], but as we have shown, constraints on the set of genotype-phenotype mappings can lead to intermediate outcomes between a single-locus polymorphism and polygenic variation where each locus has a small effect (Fig 4).

A basic question for evolutionary theory is whether evolutionary change can be seen as optimizing some form of fitness for the organism or individual [44]. Our analysis of genetic conflicts throws new light on this issue. It is reasonable to regard unlinked modifiers of effects at a polymorphic locus as representing the evolutionary interest of the organism, because unlinked, small-effect mutant modifiers share their demographic future with the organism. Our results in Fig 4—that disruptive selection can act to diminish the control exercised by unlinked modifiers over the degree of phenotypic specialization—illustrate how individual optimization might be circumvented when there is genetic polymorphism. Furthermore, our individual-based simulations with multilocus genetic architectures resulted in evolutionary outcomes that were intermediate between the evolutionary interests of linked and unlinked modifiers (Figs 4C, 4D, 5C and 5D). This fits with the general idea of the organism as a compromise between different evolutionary interests [45, 46].

In conclusion, our framework broadens the scope of social evolution theory, by accounting for adaptively maintained genetic variation in heterogeneous environments and by incorporating evolutionary outcomes over the range from genetic specialism to generalism. Many instances of interactions between relatives in nature are likely to be found somewhere between the extremes of such a spectrum. A major insight from our work is that positions along this spectrum can correspond to the degree of genetic linkage between polymorphic loci and epistatic modifiers of the phenotype in question. Our analysis thus delivers potentially testable predictions about the evolution of epistasis between modifiers and polymorphic loci and can inspire empirical investigation of the importance of genetic cues of relatedness.

## Supporting Information

S1 Text Details of model description, results and individual-based simulations. (PDF)
