# Supplementary material for: Genes as Cues of Relatedness and Social Evolution in Heterogeneous Environments
Source: PLoS Comput Biol. 2016 Jun 24;12(6):e1005006. doi: 10.1371/journal.pcbi.1005006 (PMC4920369; doi:10.1371/journal.pcbi.1005006)
Supplement: S1 Text — (PDF) [file pcbi.1005006.s001.pdf]

Supporting Information

Leimar et al., Genes as cues of relatedness and social evolution in heterogeneous environments

Detailed model description

Habitats and groups. In each of two habitats there is a large number of groups. They are formed and dissolved in a process of group turnover. A group in habitat i, where $i = 1, 2$, is founded by N_i haploid individuals, randomly derived from a pool of dispersers in that habitat. Following founding, group members reproduce asexually, forming N_i haploid offspring group members. Each founding group member has an equal chance of producing each of the N_i offspring. To study rare mutant invasion, we need the probability of identity by descent since founding of offspring group members, which is given by

$$r_i = \frac{1}{N_i}. \quad \text{(S1)}$$

This is equation (1) in the main text. To see the result, for $N_i \geq 2$, pick two random, distinct group members and note that the parent of the first has a probability of $1/N_i$ of also being the parent of the other. The purpose of the two-generation process of group formation is to implement a simple means of producing variation in relatedness. Our general approach can readily handle other, similar demographic processes whereby a group is derived from a pool of dispersers.

Social interactions. The offspring group members engage in a social interaction, for instance a public goods game, and produce offspring in proportion to the payoff in this game. An individual's phenotype z represents an investment (strategy) in the game, and we assume $0 \leq z \leq 1$. The payoff to an individual with phenotype z in habitat i is a function

$$w_i(z, \bar{z}) \quad \text{(S2)}$$

of z and the average investment $\bar{z}$ of the individual's group. For polymorphic populations the group compositions will vary. To study the invasion of a rare mutant, we need the expected payoff to a (randomly chosen) rare mutant player of the game with phenotype z', in a population where the resident phenotypes z_1 and z_2 occur with frequencies p_{i1} and p_{i2} (where $p_{i1} + p_{i2} = 1$). We write this as

$$\bar{w}_i' = \mathrm{E}[w_i(z', \bar{z})|z_1, z_2, p_{i1}, p_{i2}], \tag{S3}$$

which is equation (2) in the main text. For convenience we will sometimes also use the notation

$$\bar{w}_{ik}' = \mathrm{E}[w_i(z_k', \bar{z})|z_1, z_2, p_{i1}, p_{i2}], \tag{S4}$$

where the mutant z_k' is interpreted as a modification of z_k, as well as

$$\bar{w}_{ik} = \mathrm{E}[w_i(z_k, \bar{z})|z_1, z_2, p_{i1}, p_{i2}], \tag{S5}$$

which is the expected payoff in habitat i to the resident phenotype z_k.

The expectation in equation (S3) needs to be worked out for the particular social interaction in question, which is especially easy if $w_i(z, \bar{z})$ depends linearly on $\bar{z}$. We make use of this in a convenient example of a public goods game:

$$w_i(z, \bar{z}) = W_i + b_i \bar{z} - c_i z^2, \tag{S6}$$

where W_i is a baseline payoff, $b_i \bar{z}$ is the reward (same for all group members) of the average investment in the group, and $c_i z^2$ is the cost of the individual investment z, which is assumed to be quadratically increasing.

For a rare mutant z', founding groups that contain z' will predominantly have only one mutant individual. Based on this, we can compute a conditional expectation of the group average $\bar{z}$ as

$$\mathrm{E}[\bar{z}|z', z_1, z_2, p_{i1}, p_{i2}] = \frac{1}{N_i}\Big(z' + (N_i - 1)[r_i z' + (1 - r_i)(p_{i1} z_1 + p_{i2} z_2)]\Big), \tag{S7}$$

where the coefficient r_i of relatedness is given by equation (S1). The expected payoff in equation (S3) is then

$$\bar{w}_i' = W_i + \frac{b_i}{N_i}\Big(z' + (N_i - 1)[r_i z' + (1 - r_i)(p_{i1} z_1 + p_{i2} z_2)]\Big) - c_i z'^2. \quad \text{(S8)}$$

We use the notation d_{ik} for the derivative of this expected mutant payoff in habitat i with respect to z', evaluated at $z' = z_k$. From equation (S8) we get

$$d_{ik} = \frac{\partial \bar{w}_i'}{\partial z'}\bigg|_{z'=z_k} = \frac{b_i}{N_i}\big(1 + (N_i - 1)r_i\big) - 2c_i z_k, \quad \text{(S9)}$$

which is equation (3) in the main text.

Sequence of events in the life cycle. For simplicity, we assume that individuals are haploid over most of the life cycle. However, to explore the evolutionary consequences genetic recombination, we introduce sexual reproduction by assuming that there is a brief sexual phase in the dispersal pool in a habitat, involving diploid individuals and crossing over to produce the haploid 'asexual' individuals that found the groups as described above. Mating is random with respect to the dispersal pool and occurs before the forming of groups in the habitat. In order to have a well-defined census point, we specify the population composition at a time after the sexual phase, when groups have been formed and the public goods game is about to start. The sequence of events in the life cycle, starting right after the census point, is as follows: (*i*) public goods game with offspring production, (*ii*) within- and between-habitat migration of the offspring, forming a dispersal pool in each habitat, (*iii*) mating and recombination, and (*iv*) the next episode of group formation, including one asexual generation. An entirely asexual life cycle is a special case where the rate of recombination is zero. This is effectively the same as removing the event (*iii*) in the life-cycle.

Concerning migration, we assume that a proportion m_{ij} of the offspring formed after playing the game in habitat j join the pool of dispersers in habitat i. We might have $m_{11} = m_{22} = 1 - m$ and $m_{21} = m_{12} = m$, where m is a rate of between-

habitat migration.

Genetic cues and population dynamics of a dimorphism. We study two (or more) loci, one being a genetic cue locus with alleles with values x_k, and the other an epistatic modifier of the effects of alleles at the cue locus. We classify individuals according to their current habitat, $i = 1$ or $i = 2$, and their genetic cue, either x_1 or x_2. An individual does not directly observe the habitat, but selects a phenotype based on the genetic cue. We can regard z as a function of x as a genotype-phenotype mapping. The resident strategy is to develop phenotype z_k in response to the cue allele x_k. For this dimorphism z_1, z_2, let n_{ik} be the number of individuals in habitat i with phenotype z_k, i.e., individuals possessing the cue allele x_k at the census point. The number of individuals in habitat i is

$$n_i = \sum_k n_{ik}. \tag{S10}$$

We assume that the number of groups, g_i, that form in habitat i is constant, so the number $n_i = g_i N_i$ of individuals in a habitat at the census point is also constant. We also assume that the groups turn over in synchrony. The frequency p_{ik} of z_k in habitat i is then

$$p_{ik} = n_{ik}/n_i. \tag{S11}$$

In situations where there are no mutant alleles at modifier loci, recombination between the cue locus and modifier loci does not change the distribution of phenotypes in the dispersal pool. We can then write the resident population dynamics as

$$n_{ik}(t+1) = \sum_j \phi_i m_{ij} \bar{w}_{jk} n_{jk}(t). \tag{S12}$$

Here we used the notation

$$\phi_i = \frac{n_i}{m_{i1}(\bar{w}_{11}n_{11} + \bar{w}_{12}n_{12}) + m_{i2}(\bar{w}_{21}n_{21} + \bar{w}_{22}n_{22})}. \tag{S13}$$

We can interpret ϕ_i as the expected number of founding group members deriving

from a random individual in the pool of dispersers in habitat i (there are n_i 'openings' in habitat i and $m_{i1}(\bar{w}_{11}n_{11} + \bar{w}_{12}n_{12}) + m_{i2}(\bar{w}_{21}n_{21} + \bar{w}_{22}n_{22})$ individuals compete for these 'openings'). Note also that $\bar{w}_{jk}$ and ϕ_i in (S12, S13) depend on $n_{ik}(t)$, so the population dynamics is non-linear.

Because $n_{i1} + n_{i2} = n_i$, we can use (S12) for $k = 1$ to describe the population dynamics of a cue dimorphism:

$$\begin{aligned} n_{11}(t+1) &= \phi_1 m_{11}\bar{w}_{11}n_{11}(t) + \phi_1 m_{12}\bar{w}_{21}n_{21}(t) \\ n_{21}(t+1) &= \phi_2 m_{21}\bar{w}_{11}n_{11}(t) + \phi_2 m_{22}\bar{w}_{21}n_{21}(t). \end{aligned} \tag{S14}$$

This equation is suitable to iterate to find a population dynamical equilibrium of a cue dimorphism. Alternatively, we can use (S12) for $k = 2$ to describe the population dynamics of a cue dimorphism:

$$\begin{aligned} n_{12}(t+1) &= \phi_1 m_{11}\bar{w}_{12}n_{12}(t) + \phi_1 m_{12}\bar{w}_{22}n_{22}(t) \\ n_{22}(t+1) &= \phi_2 m_{21}\bar{w}_{12}n_{12}(t) + \phi_2 m_{22}\bar{w}_{22}n_{22}(t). \end{aligned} \tag{S15}$$

This is equivalent to (S14), but can be convenient to examine the invasion of cue allele x_2 into a monomorphism of x_1. Thus, we can use these dynamics to delineate the region in phenotype dimorphism space where z_1 and z_2, corresponding to the cue alleles x_1 and x_2, can coexist. We do this by examining when either of them can invade a monomorphism of the other. The corresponding z_1, z_2 values then form the set of possible resident dimorphisms.

Let λ^{10} be the leading eigenvalue of the 2×2 matrix described by (S15) for $n_{i2} = 0$, and thus $n_{i1} = n_i$ ($p_{i1} = 1$, $p_{i2} = 0$). This matrix describes the first order dynamics of the invasion of x_2 into a monomorphism of x_1, so x_2 can invade when $\lambda^{10} > 1$. In the same way, x_1 can invade a monomorphism of x_2 when $\lambda^{01} > 1$, where λ^{01} is the leading eigenvalue of the 2×2 matrix described by (S14) for $n_{i1} = 0$, and thus $n_{i2} = n_i$ ($p_{i1} = 0$, $p_{i2} = 1$). The region of coexistence is thus given by

$$\min(\lambda^{10}, \lambda^{01}) > 1. \tag{S16}$$

For a numerical procedure, it is convenient to have analytic expressions of these leading eigenvalues, which are readily found for 2×2 matrices.

Mutant modifier invasion. We are interested in the invasion of a mutant genotype-phenotype mapping, for which the genetic cue x_k corresponds to the phenotype z'_k, into a resident dimorphism z_1, z_2. We focus on an epistatic modifier locus where a resident allele produces the phenotype z_k when the allele x_k is present at the cue locus. Let n_{ik} denote the population dynamical equilibrium for such a resident dimorphism. For a rare mutant modifier allele, let n'_{ik} be the (small) number of mutants in habitat i that are linked to the cue allele x_k (and thus have the phenotype z'_k).

We use $(n'_{11}, n'_{21}, n'_{12}, n'_{22})$ as a vector of dynamic variables describing the mutant invasion into a resident z_1, z_2 dimorphism, and we assume that $n'_{ik} \ll n_i$. The population projection matrix $\mathbf{K}'$ is 4-dimensional, and we write its elements as $\mathbf{K}'_{ikjl}$, so that

$$n'_{ik}(t+1) = \sum_{jl} \mathbf{K}'_{ikjl} \, n'_{jl}(t), \tag{S17}$$

where

$$\mathbf{K}'_{ikjl} = \phi_i h_{ikl} m_{ij} \bar{w}'_{jl}. \tag{S18}$$

Here, h_{ikl} is the probability that a mutant modifier allele at the census point in habitat i is linked to cue allele x_k, given that its 'ancestor' at the previous census point was linked to cue allele x_l. The recombination frequency between the genetic cue and modifier loci is ρ. Considering the inheritance of modifiers, we then have

$$h_{ikl} = (1-\rho)\delta_{kl} + \rho \hat{p}_{ik} \tag{S19}$$

for the inheritance factor h_{ikl} in (S18). In equation (S19),

$$\hat{p}_{ik} = \frac{m_{i1}\bar{w}_{1k}n_{1k} + m_{i2}\bar{w}_{2k}n_{2k}}{m_{i1}(\bar{w}_{11}n_{11} + \bar{w}_{12}n_{12}) + m_{i2}(\bar{w}_{21}n_{21} + \bar{w}_{22}n_{22})} \tag{S20}$$

is the frequency of individuals in the dispersal pool in habitat i (prior to mating) that have allele x_k at the cue locus, and δ_{kl} is the Kronecker delta. Because we

assumed that the n_{ik} correspond to a resident population dynamical equilibrium, we find using (S12) that $\hat{p}_{ik} = p_{ik} = n_{ik}/n_i$. We can then write (S19) as

$$h_{ikl} = (1 - \rho)\delta_{kl} + \rho p_{ik}, \tag{S21}$$

which is is equation (9) in the main text

The population projection matrix can be written using four component 2-by-2 matrices:

$$\mathbf{K}' = \begin{pmatrix} \mathbf{K}'_{11} & \mathbf{K}'_{12} \\ \mathbf{K}'_{21} & \mathbf{K}'_{22} \end{pmatrix}. \tag{S22}$$

The components are given by

$$\mathbf{K}'_{kl} = \begin{pmatrix} \phi_1 h_{1kl} m_{11} \bar{w}'_{1l} & \phi_1 h_{1kl} m_{12} \bar{w}'_{2l} \\ \phi_2 h_{2kl} m_{21} \bar{w}'_{1l} & \phi_2 h_{2kl} m_{22} \bar{w}'_{2l} \end{pmatrix}. \tag{S23}$$

Note that the dependence on z'_l only appears in $\mathbf{K}'_{1l}$ and $\mathbf{K}'_{2l}$. For the case where the mutant is equal to the resident ($z'_k = z_k$), we use the notation

$$\mathbf{K}_{ikjl} = \phi_i h_{ikl} m_{ij} \bar{w}_{jl}. \tag{S24}$$

Invasion fitness. Given a demographic equilibrium for a resident dimorphism z_1, z_2, produced by a resident modifier of the genetic cue locus dimorphism, the invasion fitness for a mutant modifier producing z'_1, z'_2 is equal to the logarithm of the leading eigenvalue of the population projection matrix for the mutant invasion,

$$F(z'_1, z'_2; z_1, z_2) = \log \lambda, \tag{S25}$$

which is equation (4) in the main text. Here, λ is the leading eigenvalue of the matrix $\mathbf{K}'$ in (S17, S18). We know that $F(z_1, z_2; z_1, z_2) = 0$, i.e., the leading eigenvalue of the matrix $\mathbf{K}$ in (S24) is $\lambda = 1$. For an evolutionary equilibrium, we should have $F(z'_1, z'_2; z_1, z_2) \leq 0$ for any mutant z'_1, z'_2.

Details of results

Reproductive value and selection gradient. For a demographic equilibrium, with population projection matrix $\mathbf{K}'$ in (S17) with $z'_l = z_l$, so that $\mathbf{K}' = \mathbf{K}$ in (S24), we have the leading eigenvector $\boldsymbol{n} = (n_{11}, n_{21}, n_{12}, n_{22})$ and a corresponding 'left eigenvector' $\boldsymbol{v} = (v_{11}, v_{21}, v_{12}, v_{22})$ of reproductive values. Let $\boldsymbol{u}$ be the eigenvector $\boldsymbol{n}$ normalized to sum to one, i.e.,

$$u_{ik} = n_{ik}/(n_1 + n_2), \tag{S26}$$

and normalize $\boldsymbol{v}$ such that the scalar product of $\boldsymbol{v}$ and $\boldsymbol{u}$ is equal to one. We can make use of the reproductive values in the interpretation of evolutionary equilibria by noting that we can express the derivative of invasion fitness with respect to z'_l, at $z'_l = z_l$, as the matrix product

$$\left.\frac{\partial F}{\partial z'_l}\right|_{z'_l = z_l} = \boldsymbol{v}^T \left.\frac{\partial \mathbf{K}'}{\partial z'_l}\right|_{z'_l = z_l} \boldsymbol{u}. \tag{S27}$$

This follows from standard perturbation theory of operators. Note that these derivatives with respect to z'_l correspond to derivatives with respect to the modifiers of the mapping from cue to phenotype. With notation from (S9), we can use (S18) to express the mutant derivatives of the elements of the population projection matrix as

$$\left.\frac{\partial \mathbf{K}'_{ikjl}}{\partial z'_l}\right|_{z'_l = z_l} = \phi_i h_{ikl} m_{ij} d_{jl}. \tag{S28}$$

Direct fitness interpretation. The question is now if we can interpret polymorphic evolutionary equilibria as satisfying principles corresponding to the the direct fitness approach of social evolution theory. Conditional on 'observing' (being linked to) the cue allele x_k that induces z_k, a 'strategic decision maker' at a modifier locus has information about the habitat, and thus about the social

environment. The prior probability to be in habitat i is

$$q_i = \frac{n_i}{n_1 + n_2}. \tag{S29}$$

The conditional probability of being in habitat i, given the observation of the cue x_k, is then

$$q_{ik} = \Pr(i|x_k) = \frac{p_{ik}q_i}{p_{1k}q_1 + p_{2k}q_2} = \frac{p_{ik}n_i}{p_{1k}n_1 + p_{2k}n_2} = \frac{n_{ik}}{n_{1k} + n_{2k}}, \tag{S30}$$

which is equation (6) in the main text. These conditional probabilities, given a genetic cue, provide the basis for an interpretation.

From equation (S23) we see that the dependence on z_l' only occurs in the matrix components $\mathbf{K}_{1l}'$ and $\mathbf{K}_{2l}'$. Let us use the notation $\boldsymbol{v}_k = (v_{1k}, v_{2k})$ and $\boldsymbol{u}_l = (u_{1l}, u_{2l})$. Equation (S27) can be written as

$$\left.\frac{\partial F}{\partial z_l'}\right|_{z_l'=z_l} = \boldsymbol{v}_1^T \left.\frac{\partial \mathbf{K}_{1l}'}{\partial z_l'}\right|_{z_l'=z_l} \boldsymbol{u}_l + \boldsymbol{v}_2^T \left.\frac{\partial \mathbf{K}_{2l}'}{\partial z_l'}\right|_{z_l'=z_l} \boldsymbol{u}_l. \tag{S31}$$

We now have

$$\begin{aligned} &\boldsymbol{v}_k^T \left.\frac{\partial \mathbf{K}_{kl}'}{\partial z_l'}\right|_{z_l'=z_l} \boldsymbol{u}_l = \\ &\Big(v_{1k}\phi_1 h_{1kl} m_{11} + v_{2k}\phi_2 h_{2kl} m_{21}\Big) d_{1l} u_{1l} + \\ &\Big(v_{1k}\phi_1 h_{1kl} m_{12} + v_{2k}\phi_2 h_{2kl} m_{22}\Big) d_{2l} u_{2l}. \end{aligned} \tag{S32}$$

Using the notation $p_l = (n_{1l} + n_{2l})/(n_1 + n_2)$, we get that

$$\left.\frac{\partial F}{\partial z_l'}\right|_{z_l'=z_l} = \tag{S33}$$
$$\Big(v_{11}\phi_1 h_{11l} m_{11} + v_{21}\phi_2 h_{21l} m_{21} +$$
$$v_{12}\phi_1 h_{12l} m_{11} + v_{22}\phi_2 h_{22l} m_{21}\Big) d_{1l} p_l q_{1l} +$$
$$\Big(v_{11}\phi_1 h_{11l} m_{12} + v_{21}\phi_2 h_{21l} m_{22} +$$
$$v_{12}\phi_1 h_{12l} m_{12} + v_{22}\phi_2 h_{22l} m_{22}\Big) d_{2l} p_l q_{2l}.$$

We can interpret this as the conditional expectation (given the 'observation' of the cue allele x_l) of the fitness effect of a small change in z_l' away from z_l, measured in terms of reproductive value. For each observation of the cue, there are 4 possibilities of going through the population cycle, corresponding to to a combination of the outcomes of the migration and 'cue inheritance' events. Note that $p_l q_{jl} = u_{jl}$ and that p_l has the interpretation of a 'dilution factor', taking into account the the phenotypic reaction to the cue allele x_l is exposed to selection only in that part of the population that observes this cue.

Let us use the notation V_{jl} to denote the (expected) reproductive value of dispersing offspring of a player in habitat j that has observed the cue x_l, i.e.,

$$V_{jl} = v_{11}\phi_1 h_{11l} m_{1j} + v_{21}\phi_2 h_{21l} m_{2j} + \tag{S34}$$
$$v_{12}\phi_1 h_{12l} m_{1j} + v_{22}\phi_2 h_{22l} m_{2j},$$

which is equation (8) in the main text. We can now write (S33) as

$$\left.\frac{\partial F}{\partial z_l'}\right|_{z_l'=z_l} = V_{1l} d_{1l} p_l q_{1l} + V_{2l} d_{2l} p_l q_{2l}, \tag{S35}$$

which is equation (7) in the main text.

Invasion of modifier of monomorphism. We can apply the above to the

special case of a monomorphism, i.e., $z_1 = z_2 = z$, in which case we only need to keep track of the habitat i, and not the cue allele x_k, in the equations for the modifier invasion. Let n'_i be the (small) number of mutant modifier alleles in habitat i, which modify z to z'. The population projection matrix is

$$\mathbf{K}'_0 = \begin{pmatrix} \phi_1 m_{11} \bar{w}'_1 & \phi_1 m_{12} \bar{w}'_2 \\ \phi_2 m_{21} \bar{w}'_1 & \phi_2 m_{22} \bar{w}'_2 \end{pmatrix}, \tag{S36}$$

where $\bar{w}'_i$ is defined in (S3). Let us also use the notation $\bar{w}_i$, as in (S5). For $z' = z$ we have

$$\mathbf{K}_0 = \begin{pmatrix} \phi_1 m_{11} \bar{w}_1 & \phi_1 m_{12} \bar{w}_2 \\ \phi_2 m_{21} \bar{w}_1 & \phi_2 m_{22} \bar{w}_2 \end{pmatrix}, \tag{S37}$$

and one readily finds that (n_1, n_2) is a leading eigenvector of $\mathbf{K}_0$ with eigenvalue $\lambda = 1$. Note that n_i is constant in our model. We can normalize the eigenvector to (u_1, u_2) with unit sum, so that $u_i = n_i/(n_1 + n_2)$. The corresponding left eigenvector (v_1, v_2) satisfies $v_1 = v_1 \phi_1 m_{11} \bar{w}_1 + v_2 \phi_2 m_{21} \bar{w}_1$, so that

$$\frac{v_1}{v_2} = R_{12} = \frac{\phi_2 m_{21} \bar{w}_1}{1 - \phi_1 m_{11} \bar{w}_1}, \tag{S38}$$

where we introduced the notation R_{12} for the ratio. Using the normalization $v_1 u_1 + v_2 u_2 = 1$ for the reproductive values, we have

$$\begin{aligned} v_1 &= \frac{R_{12}}{R_{12} u_1 + u_2} \\ v_2 &= \frac{1}{R_{12} u_1 + u_2}. \end{aligned} \tag{S39}$$

Just as for the dimorphism case, we can write the selection gradient as

$$\left. \frac{\partial F}{\partial z'} \right|_{z'=z} = \boldsymbol{v}^T \left. \frac{\partial \mathbf{K}'_0}{\partial z'} \right|_{z'=z} \boldsymbol{u}, \tag{S40}$$

where $\boldsymbol{v} = (v_1, v_2)$ and $\boldsymbol{u} = (u_1, u_2)$. Introducing

$$d_i = \left.\frac{\partial \bar{w}_i'}{\partial z'}\right|_{z'=z}, \tag{S41}$$

and using the vector of reproductive values from (S39), the selection gradient at the monomorphism z is then

$$\left.\frac{\partial F}{\partial z'}\right|_{z'=z} = \big(v_1\phi_1 m_{11} + v_2\phi_2 m_{21}\big) d_1 u_1 + \big(v_1\phi_1 m_{12} + v_2\phi_2 m_{22}\big) d_2 u_2. \tag{S42}$$

This expression can be used to compute monomorphic equilibria numerically.

Numerical approach.

The evolutionary equilibria shown in Fig. 2 and Fig. 3 in the main text were computed numerically using the selection gradient from equation (7) in the main text. For this computation, we developed a C++ program that follows a path of small steps through z_1z_2–space, each of which increase the invasion fitness in equation (4) in the main text, until reaching an accurate approximation of the equilibrium. The location of monomorphic equilibria, illustrated in Fig. 2 in the main text, was similarly determined using the selection gradient (S42). The coexistence regions in Fig. 3 in the main text were computed numerically using the approach given by equation (S16). The C++ program is available from the corresponding author upon request.

Individual-based simulations

In all simulations we used the genotype-phenotype mapping

$$z = \frac{1}{1 + \exp(-a_0 - a_g x)}, \tag{S43}$$

which is equation (5) in the main text. Here $a_0 + a_g x$ is a ‘liability’ to develop the phenotype z given the cue x, with parameters a_0 and a_g. The genetic cue was

always encoded by a single locus, but the parameters a_0 and a_g had different genetic architecture in different simulations. Our general approach was to develop C++ programs that directly implemented the sequence of events in the life cycle, using pseudo-random numbers to handle stochastic events, such as recombination and mutation. Alleles were encoded as real numbers, restricted to a particular range for each locus. Mutation of an allele was implemented as a random increment to the allelic value, having a bi-exponential distribution with a particular standard deviation for each locus. In case a mutated value exceeded the range of a locus, the value was set to the corresponding limit of the range. For the results we report, simulations were run for 20000 full life cycles with a rate of mutation of 0.0010, to generate variation, followed by another 20000 full life cycles with a smaller rate of mutation of 0.0001. We used a total population size of 40000, which is fairly large, but there is still some random variation in average phenotypes from simulation to simulation. We illustrate this variation in figures by showing the mean $\pm$ SD over several replicate simulations.

For the simulations reported in Fig. 2 and Fig. 4 *A* and *B* in the main text there were 3 loci: a genetic cue locus coding for x and one locus for each of a_0 and a_g. The latter two loci were fully linked, and their rate of recombination with the cue locus was ρ. The range of allelic effects was $[-1, 1]$ at the cue locus, $[-2, 2]$ at the locus for a_0 and $[0, 5]$ at the locus for a_g. In Fig. 2 in the main text, evolutionary change at the locus for a_0 was prevented by setting the mutational increments to zero for this locus. The allelic value at the locus was initialized to a (fixed) value that would allow evolutionary adjustments in a_g and a polymorphism in x to closely approximate the values obtained from setting the selection gradient, given by [7] in the main text, to zero. These fixed values were as follows. For $\rho = 0$ and $m = 0.01, 0.05, 0.10$ we used $a_0 = -0.5185168, -0.3610248, -0.3082787$, and for $\rho = 0.5$ and $m = 0.01, 0.05, 0.10$ we used $a_0 = -0.5625004, -0.5375327, -0.4446206$. The purpose of this approach, with suitable fixed values of a_0, is to illustrate simulation outcomes where there is little scope for genetic conflict to produce polymorphism at modifier loci. Different fixed values of a_0 essentially correspond to different

genetic constraints. On the other hand, for the simulations in Fig. 4 *A* and *B* in the main text, effects at the locus for a_0 were free to evolve, resulting in the same outcome in some cases, but also in the transfer of polymorphism to the locus for a_0, as illustrated in Fig. 4 *B* in the main text.

For the simulation shown in Fig. 4 *C* in the main text, the genetic cue locus had effects with a range of $[0, 1]$. The parameter a_0 was the sum of positive effects at 5 loci, each with a range of $[0, 0.4]$, and negative effects at another 5 loci, each with a range of $[-0.4, 0]$. The parameter a_g was the sum of positive effects at 10 loci, each with a range of $[0, 0.3]$. All loci were unlinked.

For the simulation shown in Fig. 4 *D* in the main text, the genetic cue locus had effects with a range of $[0, 1]$ and the parameter a_g was the sum of positive effects at 10 loci, each with a range of $[0, 0.3]$. However, the parameter a_0 had a more complex genetic architecture. There was a sum of effects at 5 loci with a range of $[0, 0.4]$, and a sum of effects at another 5 loci with a range of $[0, 0.4]$, but these sums of effects were each in turn controlled by another locus that put a ceiling on the maximum total expression. So if ξ would be the the total expression without a ceiling ξ_{max}, then the expression was η with the ceiling, where η is given as follows:

$$\eta = \xi_{\text{max}}\big(1 - \exp(-\xi/\xi_{\text{max}})\big). \tag{S44}$$

This is meant to correspond to a situation where a regulatory locus limits the expression at other loci. The parameter a_0 was the difference between these two regulated sums of effects. Again, all loci were unlinked in the simulation.

The C++ source code for the simulation programs is available from the corresponding author upon request.
